# Primary care anti-cyclic citrullinated protein antibody testing for rheumatoid arthritis and accompanying pathway redesign reduces unnecessary referrals

**DOI:** 10.1093/rap/rkag103

**Published:** 2026-08-17

**Authors:** George F G Allen, Timothy J McDonald, Stuart Kyle, Nick Keysell

**Affiliations:** NIHR Exeter Biomedical Research Centre, University of Exeter, Exeter, UK; Blood Sciences, Royal Devon University Healthcare NHS Foundation Trust, Exeter, UK; NIHR Exeter Biomedical Research Centre, University of Exeter, Exeter, UK; Blood Sciences, Royal Devon University Healthcare NHS Foundation Trust, Exeter, UK; Rheumatology, Royal Devon University Healthcare NHS Foundation Trust, Barnstaple, UK; Devon Referral Support Service, NHS Devon, Exeter, UK; Clare House Surgery, Tiverton, UK

**Keywords:** ACPA, anti-CCP, inflammatory arthritis

## Abstract

**Objectives:**

Rheumatoid factor (RF) and anti-cyclic citrullinated peptide (anti-CCP) antibodies are alternative diagnostic tests for RA with similar sensitivity. Anti-CCP antibodies have improved specificity compared with RF and so should result in fewer false positives. A retrospective, observational study was conducted following a change in the primary care pathway and test for inflammatory arthritis from RF to anti-CCP antibodies, assessing real-world impact on referrals.

**Methods:**

The primary care test was changed from RF to anti-CCP antibodies as part of a service development in East and North Devon, UK (population 463 000). Data were monitored post-implementation from 1 March to 31 August 2024. Anti-CCP antibody testing was made available to primary care. General practitioner order communications automatically redirected requests for RF to anti-CCP antibodies. Local referral guidelines were implemented recommending anti-CCP antibodies to support referral decisions but not for rule-out of inflammatory arthritis. The equivalent 6-month period from 2023 when RF was the primary test for inflammatory arthritis was used for comparison.

**Results:**

Positive tests decreased from 10.6% with RF to 3.9% with anti-CCP antibodies. This led to 57 (39%) fewer referrals following a positive test. The false positive rate among those referred to rheumatology decreased from 59% (91/148) with RF to 32% (29/91) with anti-CCP antibodies. Anti-CCP antibodies and RF detected similar numbers of inflammatory arthritis cases with a case yield of 2.1% for both tests.

**Conclusion:**

Replacement of RF with anti-CCP antibodies within a redesigned inflammatory arthritis pathway reduced unnecessary rheumatology referrals. This could help oversubscribed services reduce waiting times and thus contribute to timely diagnosis of inflammatory arthritis.

Key messages

- Using anti-cyclic citrullinated protein (anti-CCP) antibodies instead of RF in primary care reduces unnecessary referrals.
- Anti-CCP antibodies reduced false positive referrals compared with RF.
- The number of cases detected remained unchanged with either anti-CCP antibodies or RF.

## Introduction

Anti-cyclic citrullinated protein (anti-CCP) antibodies and rheumatoid factor (RF) are alternative blood tests with similar sensitivity for RA but anti-CCP antibodies has higher specificity [1]. A meta-analysis of diagnostic accuracy studies found similar pooled sensitivity for RA of 67% for anti-CCP antibodies and 69% for RF [1], indicating that both detect a similar number of cases. However, pooled specificity was higher for anti-CCP antibodies at 95% compared with 85% for RF. Therefore the anti-CCP antibodies false positive rate will be lower.

Diagnostic criteria for RA include RF and anti-CCP antibodies as alternative tests, but the UK National Institute for Health and Care Excellence (NICE) recommends RF as the first-line test for RA in primary care [2, 3]. The ACR and EULAR RA diagnostic classifications include anti-CCP antibodies or RF positivity as equally weighted criteria [3]. NICE NG100 recommends RF in primary care for those with suspected RA and clinical synovitis and to consider anti-CCP antibody testing only if RF is negative [2]. A lack of evidence on cost-effectiveness prevented recommendations for wider use of anti-CCP antibodies [4].

On the basis of similar sensitivity but a likely reduction in false positive results, we replaced RF with anti-CCP antibodies in primary care within a redesigned referral and triage pathway. We performed a retrospective, observational study to evaluate the effect on referrals and real-world test positivity rate but not diagnostic accuracy.

## Methods

### Ethics

This service development was approved by the Peninsula Pathology Effectiveness Group, a regional panel with representation from four acute hospital trusts and primary care, with authority to implement changes to patient pathways and monitor outcomes.

### Location and population

The service change was undertaken at primary care practices served by Royal Devon University Healthcare NHS Foundation Trust. This area approximates to the regions of North, Mid and East Devon, UK ,which had a population of 463 000 at the last national census on 21 March 2021 [5].

### Tests

Serum anti-CCP antibodies were analysed using an Elecsys Anti-CCP electrochemiluminescence immunoassay on a Cobas e801 analyser (Roche, Basel, Switzerland). Serum RF was analysed using a Roche RF-II immunoturbidimetry assay on a Cobas 501 analyser. The pooled mean coefficient of variance during the observation period was 4.1% for anti-CCP antibodies and during the comparative period for RF was 2.2%. The cut-off for positive values was 17 U/ml for anti-CCP antibodies and 14 IU/ml for RF as defined by the manufacturer.

### Service changes

The general practice electronic order communications system was updated to make anti-CCP antibodies available to order. RF was made unavailable and test requests for RF were redirected to anti-CCP antibodies. A positive anti-CCP antibodies result led to automatic testing of RF on the primary sample to ensure this was available for later rheumatology review. Local referral guidelines for inflammatory arthritis were updated to recommend anti-CCP antibodies instead of RF for patients with musculoskeletal symptoms where a diagnosis of inflammatory arthritis was being considered (see Supplementary Data S1). Referral was recommended without waiting for test results if the clinician was confident in a diagnosis of inflammatory arthritis.

### Data extraction and analysis

Testing and referral data were extracted from electronic patient records (EPIC systems). Test data with anti-CCP antibodies as the primary test were extracted from 1 March 2024 to 31 August 2024 and the comparative period with RF from 1 March 2023 to 31 August 2023. Referral data were extracted from 1 March to 30 September for respective years to allow an additional 1 month for referral. A total of 10 patients with positive RF and 3 with positive anti-CCP antibodies were referred prior to testing and so were excluded from referral and outcome data. True cases were defined as patients with a diagnosis of inflammatory arthritis following a rheumatology referral. Positive test results were classified as false positive if the clinical diagnosis following referral was not inflammatory arthritis or the referral was rejected by a rheumatologist, i.e. they assessed remotely that the patient did not have inflammatory arthritis. Referrals were rejected on the basis of clinician judgement and triage decision-making without further assessment to confirm categorisation as false positive. For this reason, a sensitivity analysis was performed to confirm that inclusion of rejected referrals did not alter interpretation of our findings. Where stated, results were standardised to mean test number between the two observation periods. Group differences were assessed using chi‑squared tests. Effect sizes were expressed as risk ratios (RRs) with 95% CIs. Data were analysed in R [6–9] (code available at https://github.com/gfgallen3/ACPA/).

## Results

### Fewer positive tests with anti-CCP antibodies compared with RF

**Figure 1:**
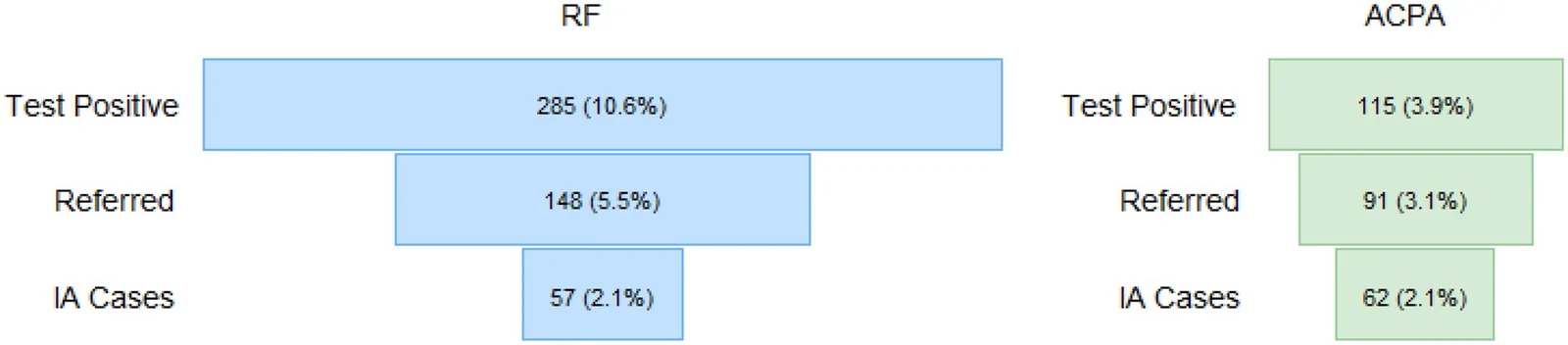
Fewer referrals with anti-CCP antibodies compared with RF but similar case detection. ACPA: anti-cyclic citrullinated protein antibodies; IA: inflammatory arthritis. Inflammatory arthritis cases determined from diagnosis following rheumatologist clinical review. Total number of tests over a 6-month observation period with RF was 2696 and with ACPA was 2932

In the 6-month period with RF as the primary test for inflammatory arthritis, 2696 tests were ordered in primary care and 285 (10.6%) of these were positive (see Fig. 1). In the 6-month period with anti-CCP antibodies as the primary test, the number of positive tests decreased to 115 (3.9%) of the 2932 tests ordered [RR 0.930 (95% CI 0.917, 0.944), χ^2^(1) = 94.83, *P* < 0.0001].

### Fewer referrals following a positive test with anti-CCP antibodies compared with RF

With RF there were 148 referrals following a positive test (5.5% of patients tested; see Fig. 1). With anti-CCP antibodies this decreased to 91 referrals following a positive test [3.1% of patients tested, RR 0.975 (95% CI 0.964, 0.986), χ^2^(1) = 19.93, *P* < 0.0001]. Using a standardised number of tests to allow direct comparison, changing from RF to anti-CCP antibodies would result in 134 fewer rheumatology referrals following a positive test per year (see Supplementary Table S2).

### A similar number of inflammatory arthritis cases were detected with RF and anti-CCP antibodies

From those who were referred to rheumatology following a positive RF test, 57 of 148 had a subsequent diagnosis of inflammatory arthritis (see Fig. 1). With anti-CCP antibodies, inflammatory arthritis was diagnosed in 62 of 91. This gives the same case yield from tests performed in the 6-month periods of 2.1% for RF and 2.1% for anti-CCP antibodies.

### Fewer false positives with anti-CCP antibodies compared with RF

With RF as the primary test, 59% (91/148) of cases reviewed by a rheumatologist either remotely or in clinic were false positive, i.e. not diagnosed with inflammatory arthritis. With anti-CCP antibodies this decreased to 32% (29/91) false positive [RR 0.565 (95% CI 0.441, 0.724), χ^2^(1) = 19.77, *P* < 0.0001]. The positive predictive value of RF was 20% and anti-CCP antibodies was 54%. Of those not diagnosed with inflammatory arthritis, an alternative significant rheumatological condition was diagnosed in three patients with RF and one patient with anti-CCP antibodies (see Supplementary Table S1).

A sensitivity analysis was performed by excluding all rejected referrals to ensure their inclusion in the definition of false positive did not influence the outcome of the anti-CCP antibodies to RF comparison. With rejected referrals excluded, a reduction in false positives with anti-CCP antibodies remained, where 53% (63/120) were false positive with RF and 29% (25/87) with anti-CCP antibodies [RR 0.667 (95% CI 0.529, 0.839), χ^2^(1) = 11.65, *P* = 0.0006].

### Minimal impact of anti-CCP antibodies on referrals with a negative test and an overall net benefit of reduced referrals

With RF, 17.1% (461/2696) of those tested were referred to rheumatology with a negative test. With anti-CCP antibodies, 17.5% (514/2932) were referred with a negative test, confirming neither test was being used to rule out inflammatory arthritis and general practitioners felt confident referring patients with negative RF or anti-CCP antibodies. Using a standardised number of tests for direct comparison, changing from RF to anti-CCP antibodies would result in an increase of 20 referrals per year among those with a negative test result (see Supplementary Table S2). Including patients with positive and negative test results, the net benefit of changing the test from RF to anti-CCP antibodies was a 9% (114/1271) reduction in referrals for patients who have had a serum test for inflammatory arthritis in primary care (see Supplementary Table S2).

## Discussion

Using anti-CCP antibodies as the primary supportive test in a redesigned inflammatory arthritis referral and triage pathway led to a reduction in unnecessary referrals to rheumatology. This has the potential to reduce waiting times for oversubscribed services and thus improve timely diagnosis and treatment of inflammatory arthritis. The number of positive tests decreased from 10.6% with RF to 3.9% with anti-CCP antibodies, which led to a 39% reduction in rheumatology referrals following a positive test. Crucially for those referred to rheumatology, the false positive rate decreased with anti-CCP antibodies and a similar number of cases were detected.

It is important to highlight that anti-CCP antibodies were not used to rule out inflammatory arthritis in our pathway. Anti-CCP antibodies were recommended to support referral decisions in those with suspected inflammatory arthritis. A positive result was not required for referral, as criteria were clear to refer if symptoms and signs suggested inflammatory arthritis. The sensitivity of anti-CCP antibodies and RF for RA is relatively low [1] and other forms of inflammatory arthritis, such as seronegative RA or PsA, present without anti-CCP antibodies or RF serum positivity.

The improved specificity of anti-CCP antibodies over RF is well established; however, RF has an independent role in prognosis [1, 10, 11] that was accounted for in our testing pathway by including automatic addition of RF to all samples with positive anti-CCP antibodies. This led to 115 RF tests from 2932 anti-CCP antibody requests, which was 0.4% of the total cost of anti-CCP antibody testing. This ensured that both markers were available at referral to enable clinical review and prognostication. Both anti-CCP antibodies and RF are associated with progressive joint damage and extra-articular manifestations, while RF is associated with disease activity and dual RF and anti-CCP antibody positivity are associated with more rapid progression [10–15].

Our data suggest that the use of anti-CCP antibodies instead of RF could reduce costs within the patient pathway despite higher laboratory costs. Laboratory costs are typically greater for anti-CCP antibodies than RF [16] and we anticipate additional costs of ≈£11 000 per annum for our laboratory. We speculate that increased laboratory costs could be offset by cost reductions associated with unnecessary rheumatology referrals. However, this would require a health economic evaluation to confirm cost-effectiveness, which was beyond the scope of our service development. Konnopka *et al.* [17] found that anti-CCP antibodies were likely to be cost-effective for early diagnosis of RA but there was uncertainty regarding the cost impact of later diagnosis. Cost-effectiveness studies are lacking and, in particular, there is no published evidence on cost-effectiveness of anti-CCP antibodies *vs* RF within primary care [4].

The strength of our data is that it was collected as part of a real-world routine service development and was efficiently captured via the hospital’s electronic patient record. As a report of a routine service change, our data demonstrate the real-world effect of changing a primary care pathway for inflammatory arthritis that included using anti-CCP antibodies instead of RF. The electronic patient record allowed for effective and complete collection of laboratory and referral data for all patients referred to rheumatology at our hospitals, minimising the risk of patients missing from follow-up.

The main limitations of the reported data are that comparisons were retrospective, data were not collected as part of a randomised controlled trial, additional changes beyond test substitution were made and there was a short follow-up period for referrals (minimum 1 month). Comparisons were made before and after the change in practice rather than by direct comparison or a randomised controlled trial, which would be required to determine a causal effect. There may have been systematic differences beyond the test change between the time periods that could influence the number of referrals. Time periods were matched to minimise seasonal variation.

The referral guidance and test ordering system were also modified. These changes may have impacted referrals through changes to clinical practice beyond test substitution, such as influencing confidence in referral decisions or raising clinical awareness. The addition of RF to positive anti-CCP antibodies could potentially have biased the comparison to the RF period where rheumatologist review would have included RF only. Our definition of false positive included rejected referrals, which were rejected on the basis of referral and triage decisions alone without further assessment that would confirm the absence of disease. However, we performed a sensitivity analysis with all rejected referrals excluded and there was still a significant reduction in false positive results with anti-CCP antibodies compared with RF.

In conclusion, the use of anti-CCP antibodies in place of RF in primary care as part of a redesigned referral pathway reduces the number of patients unnecessarily referred to rheumatology for suspected RA. Changing the test from RF to anti-CCP antibodies thus has the potential to make more efficient use of rheumatology services, decrease waiting lists and improve timely review of patients.

## Supplementary Material

rkag103_Supplementary_Data

## Data Availability

The data underlying this article are derived from routine healthcare records and therefore cannot be shared publicly.
