# Supplementary material for: Primary care anti-cyclic citrullinated protein antibody testing for rheumatoid arthritis and accompanying pathway redesign reduces unnecessary referrals
Source: Rheumatol Adv Pract. 2026 Aug 17;10(3):rkag103. doi: 10.1093/rap/rkag103 (PMC13546031; doi:10.1093/rap/rkag103)
Supplement: Supplementary file 1 [file rkag103_supplementary_data.docx]

Supplementary Data S1

The following referral guidance text was written by Stuart Kyle and Nick Keysell on behalf of NHS Devon and was originally published February 2024 on the following webpage: https://northeast.devonformularyguidance.nhs.uk/referral-guidance/eastern-locality/rheumatology/suspected-inflammatory-arthritis-pathway-north-and-east-devon.

**Suspected Inflammatory Arthritis Pathway – North and East Devon**

**Key Messages**

Early recognition & treatment of inflammatory arthritis significantly improves outcome. Delay reduces efficacy of treatment and leads to poor outcome. After consultant triage, if thought suitable then patients will be seen in ‘The Early Arthritis Clinic’ within 2-3 weeks.

**Please** DO NOT **administer steroid before referral & assessment in clinic unless discussed with rheumatology team.**

Please refer to your local rheumatology service – these patients require lots of visits, tests & monitoring, they should be seen where their acute care would be best placed.

See referral section for the contact details for any urgent rheumatology queries.

**Assessment**

**The following are clues to an Inflammatory Arthritis – Please see specific criteria for Referral to Early Arthritis further down page**

**History**

- Prolonged morning stiffness (more than 30mins), response to NSAID, & joint swelling.
- Extra-articular features: Psoriasis, rheumatoid nodules, sicca (dry eyes and mouth), inflammatory eye disease, STI/urethritis/gastroenteritis.
- Risk Factors: family history & smoking (RA).

**Examination**

- Symmetry, small joint synovial (soft tissue) swelling.
- Presence of enthesitis e.g., sausage digit, Achilles tendinitis.
- Distribution : e.g., wrists, MCP & PIP and feet MTP for rheumatoid arthritis.
- Look for extra-articular features

**Differential Diagnoses**

Include:

- Septic arthritis
- Crystal arthropathy (gout & pseudogout)
- Osteoarthritis
- Perimenopause
- Post viral arthritis
- Sarcoidosis
- Fibromyalgia

**Red Flags**

The S Factor? (used by RCGP & NRAS)

- Stiffness - Early morning joint stiffness lasting more than 30 minutes
- Swelling - Persistent swelling of one joint or more, especially MCP joints
- Squeezing - Squeezing the joints is painful in inflammatory arthritis

**Investigations**

- aCCP, FBC, U&E, LFT, CRP, urate

*If confident of an inflammatory arthritis (IA) diagnosis, please refer before waiting on blood results.*

aCCP

- aCCP is a newer and more specific investigation for RA (thus greatly reducing the false positive rates and patient anxiety when compared to RhF)
- aCCP should be used when considering a diagnosis of RA in patients with MSK symptoms

Rheumatoid factor (RhF)

- RhF should no longer be requested routinely in primary care.
- RhF is useful for prognosis, predicting complications and guiding management in secondary care.
- aCCP should be used when investigating suspected early inflammatory arthritis. If aCCP is positive, RhF will be automatically requested by the lab.

CRP

- this can be very helpful in supporting clinical signs and symptoms of inflammatory arthritis.
- Please note: patients in whom IA is clinically suspected should be referred even with a normal CRP.
- CRP can be mildly raised in patients with elevated BMI. In the absence of clinical signs and symptoms of inflammatory arthritis, a raised CRP alone should not be an indication for referral

Urate

- Is not diagnostic and may be falsely normal in an acute attack of gout
- Serum urate levels should be checked 4-6 weeks after an acute attack
- The main use of urate is in titrating prophylactic treatments e.g., allopurinol

ANA

- only indicated if the patient has other features of autoimmune disease .

HLA-B27

- please only check HLA-B27 if advised by secondary care

X-Rays

- X-rays will be requested by **secondary care** as indicated and are NOT required prior to referral.

**Management**

Please see the Formulary section on Musculoskeletal & joint diseases:

[North & East Formulary](https://northeast.devonformularyguidance.nhs.uk/formulary/chapters/10.-musculoskeletal-joint-diseases)

Please **DO NOT** administer steroid before referral & assessment in clinic unless discussed with rheumatology team.

If in doubt, please contact your local rheumatology team for advice. See referral section for the contact details for any urgent rheumatology queries.

**Referral**

**Referral criteria**

1. Suspicion inflammatory Arthritis as above

2. Symptoms more than 4 weeks & less than 6months*

3. Not explained by flare of osteoarthritis / gout

*Patients with longer history will be seen in general rheumatology clinic

**Referral instructions**

1. Request *early inflammatory arthritis clinic* via DRSS referral on e-RS

2. Include duration of symptoms & examination findings - very important for triage

3. Please refer to your local rheumatology service – these patients require lots of visits, tests & monitoring, they should be seen where their acute care would be best placed

Supplementary Table S1. Diagnoses of patients referred following a positive test result.

| **Category** | **Diagnosis** | **ACPA** | **RF** |
| --- | --- | --- | --- |
| False Positive | Referral rejected by rheumatologist | 4 | 28 |
|  | Osteoarthritis | 9 | 26 |
|  | Joint pain | 7 | 9 |
|  | Fibromyalgia | 2 | 5 |
|  | Diagnosis unknown | 0 | 1 |
|  | Arthralgia | 3 | 8 |
|  | Reactive arthritis | 0 | 1 |
|  | Back pain | 1 | 1 |
|  | Trochanteric bursitis | 0 | 1 |
|  | Carpal tunnel | 0 | 1 |
|  | Gout | 0 | 2 |
|  | Raynaud | 0 | 1 |
|  | Sicca syndrome | 0 | 1 |
|  | Swollen finger | 0 | 1 |
|  | Pseudogout | 0 | 1 |
|  | Torticollis | 0 | 1 |
|  | Hand pain | 1 | 0 |
|  | Neck pain | 1 | 0 |
| False Positive (significant rheumatological condition) | Polymyalgia rheumatica | 1 | 2 |
|  | Connective tissue disease | 0 | 1 |
| Inflammatory Arthritis | Rheumatoid arthritis | 55 | 50 |
|  | Other inflammatory arthritis | 1 | 4 |
|  | Psoriatic arthritis | 3 | 2 |
|  | Ankylosing spondylitis | 0 | 1 |
|  | Palindromic rheumatism | 2 | 0 |
|  | Monoarthritis | 1 | 0 |

ACPA: Anti-cyclic Citrullinated Protein Antibodies. RF: Rheumatoid Factor

Supplementary Table S2. Calculation of standardised, annualised referrals and net benefit. ACPA: Anti-cyclic Citrullinated Protein Antibodies.

| **Positive test results** | **RF** | **ACPA** | **Mean** |
| --- | --- | --- | --- |
| Total tests | 2696 | 2932 | 2814 |
| Referrals | 148 | 91 | NA |
| Proportion referred (referrals/total tests) | 0.055 | 0.031 | NA |
| Referrals standardised to mean total tests (proportion referred*mean total tests) | 154.5 | 87.3 | NA |
| Difference in standardised referrals (RF – ACPA) | NA | -67 | NA |
| Positive test annualised difference (6 month period*2) | NA | -134 | NA |
| **Negative test results** |  |  |  |
| Total tests | 2696 | 2932 | 2814 |
| Referrals | 461 | 512 | NA |
| Proportion referred (referrals/total tests) | 0.171 | 0.175 | NA |
| Referrals standardised to mean total tests (proportion referred*mean total tests) | 481.2 | 491.4 | NA |
| Difference in standardised referrals (RF – ACPA) | NA | 10 | NA |
| Negative test annualised difference (6 month period*2) | NA | 20 | NA |
| **Net benefit** |  |  |  |
| Standardised and annualised total no. referrals (positive + negative standardised referrals)*2 | 1271 | 1157 |  |
| Net benefit (no. tests, positive + negative test annualised difference) | NA | -114 | NA |
| % net benefit (net benefit/standardised total no. referrals RF)*100 | NA | -9 | NA |

RF: Rheumatoid Factor. NA: Not Applicable. Data was collected over a 6-month period and so where indicated was annualised by multiplying by 2.
